# Supplementary material for: Artificial Intelligence in Decrypting Cytoprotective Activity under Oxidative Stress from Molecular Structure
Source: Int J Mol Sci. 2023 Jul 12;24(14):11349. doi: 10.3390/ijms241411349 (PMC10379162; doi:10.3390/ijms241411349)
Supplement: Supplementary file 1 [file ijms-24-11349-s001.zip › Files.pdf]

Files

The Files are stored on GitHub website, and can be accessed via the link: https://github.com/XDamianX-coder/Indole_new_structures

File S1.	Indole_-_cytoprotekcja_.xlsx
File S2.	Training_data_preparation.ipynb
File S3.	test_training_data_smiles.parquet
File S4.	Neural_network.ipynb
File S5.	Prediction_1_0.1_tensor_noise.ipynb
File S6.	Prediction_1_0.2_tensor_noise.ipynb
File S7.	Molecules_generated_tensor_scaling_0_1.xlsx
File S8.	Molecules_generated_tensor_scaling_0_2.xlsx
File S9.	Predictive_model-Decision_Tree-Train.ipynb
File S10.	Predictive_model-KNeighbors.ipynb
File S11.	Predictive_model-Random_forest.ipynb
File S12.	Predictive_model-SVM.ipynb
File S13.	Predictive_model.ipynb
File S14.	create_whole_report.py
File S15.	Whole_report.xlsx
File S16.	gramina_tiouracyle_pred_pic.xlsx
File S17.	Generated_structures_PubChem_search.ipynb
File S18.	new_structures_indole.xlsx
File S19.	Tanimoto_similarity_report.ipynb
File S20.	Proposed_structures_with_AI_indole_tanimoto_similarity_.xlsx
File S21.	Tanimoto_similarity_pictures.ipynb
File S22.	SYBA_selected_structures_similarity.xlsx
File S23.	t-SNE_chemical_space.ipynb
File S24.	The_final_regression_predictive_model_decision_tree_scheme.pdf
File S25.	Additional_metrics_of_AI.ipynb
