# Supplementary material for: Artificial Intelligence in Decrypting Cytoprotective Activity under Oxidative Stress from Molecular Structure
Source: Int J Mol Sci. 2023 Jul 12;24(14):11349. doi: 10.3390/ijms241411349 (PMC10379162; doi:10.3390/ijms241411349)
Supplement: Supplementary file 1 [file ijms-24-11349-s001.zip › in vitro Biological assays.pdf]

The structure of compounds tested in *in vitro* assays

compound 1

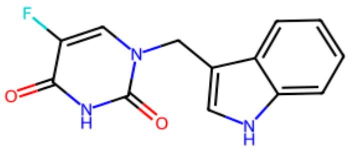

compound 2

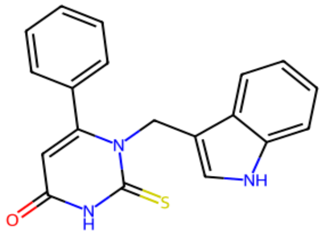

compound 3

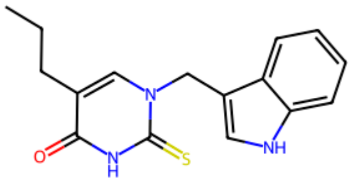

compound 4

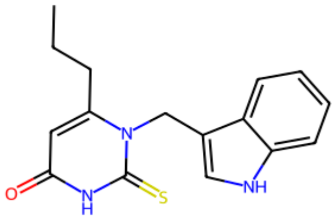

Biological activity *in vitro* – assays, results, main conclusion, material and methods

1. Hemolytic activity (human red blood cells, RBC)

- after the standard incubation (1 hour at 37°C) at the concentration of 0.05 and 0.1 mg/mL, compounds 1-4 are cytotoxic (hemolysis degree >5%, Table S1); hemolytic activity of all compounds is a structure- and a dose-dependent

- after the standard incubation (1 hour at 37°C) at concentration of 0.01 mg/mL hemolytic activity of compounds (Table S1) is higher than obtained for negative control (PBS buffer) equal to **3.14±0.14** (Fig. S5); however, any value lower than 5% allows any compounds defined as hemocompatible for potential biomedical applications.

Table S1

Hemolytic activity (%) of compounds 1-4 at the concentration range from 0.1 mg/mL to 0.01 mg/mL presented as mean ± SD (n=11-17). after standard incubation (1 hour, 37°C) Hemolysis for negative control (PBS buffer) was equal to **3.14 ± 0.14**

Compound	0.1 mg/mL	0.05 mg/mL	0.01 mg/mL

1	9.57 ± 3.99	5.60 ± 2.09	4.13 ± 1.13
2	16.71 ± 6.65	10.97 ± 3.98	4.89 ± 1.24
3	17.36 ± 6.60	8.44 ± 2.47	4.84 ± 0.84
4	8.79 ± 2.21	4.92 ± 0.45	4.34 ± 0.54

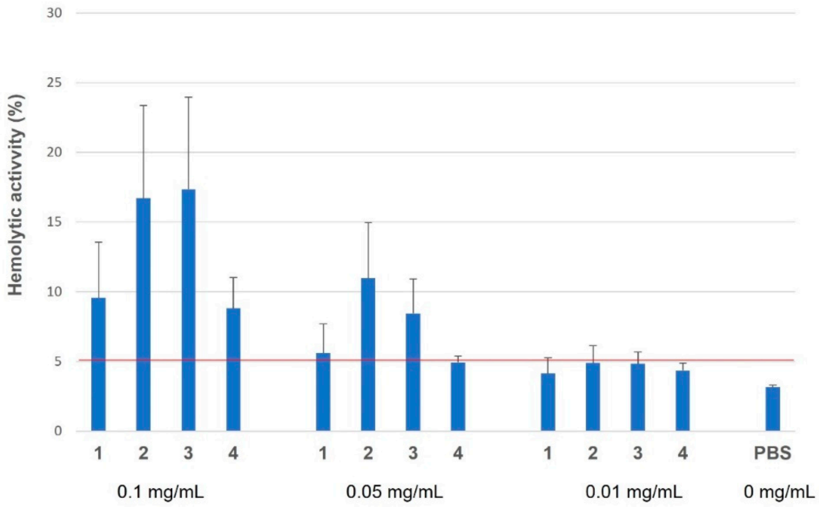

Fig. S5. Hemolytic activity (%) of compounds 1-4 at different concentrations (mg/mL) after 1 hour incubation at 37°C. Hemolysis calculated for a negative control (RBC in PBS buffer, 0 mg/mL) is also presented. The red line is plotted for a hemolysis threshold of 5%. Hemolysis activity higher than 5% indicates a membrane-perturbing activity of the compound and its cytotoxic properties. Values are presented as mean ± SD (n=11-17)

2. Cytoprotective activity under oxidative stress conditions

All compounds inhibit an oxidative hemolysis induced by free radical generated from the standard oxidative hydrofilc agent 2,2'-azobis(2-methylpropionamidine) dihydrochloride (AAPH) in the structure-depended manner (Fig. S6). The cytoprotective activity of compounds 3 and 4 is statistically the same (p>0.05) as obtained for the Trolox (standard antioxidant). The chemical structure of 3 and 4 is similar (prediction – *the role of the propyl groups in the specific membrane incorporating*).

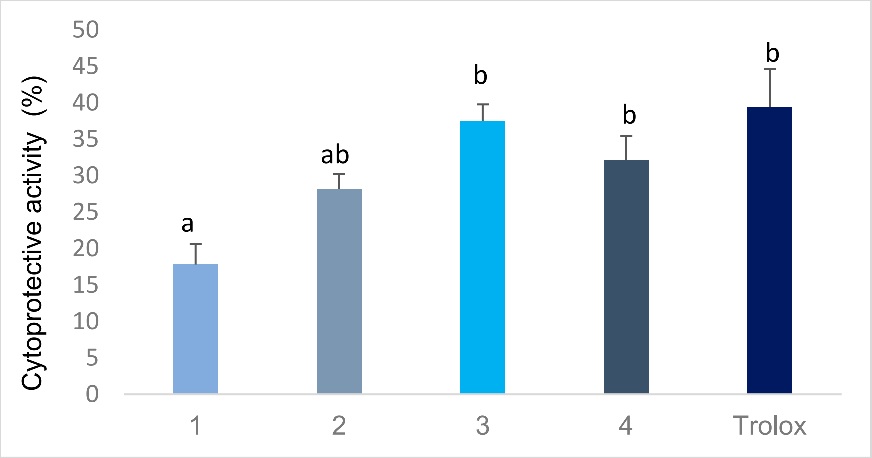

APPH

Tukey HSD[a,b]

Sample	N	Subset for alpha = 0.05	
		1	2
1	12	17.81113040	
2	13	28.20196756	28.20196756
4	13		32.15883628
3	13		37.51416702
Trolox	12		39.42222935
Sig.		.096	.060

Means for groups in homogeneous subsets are displayed.

a. Uses Harmonic Mean Sample Size = 12.581.

b. The group sizes are unequal. The harmonic mean of the group sizes is used. Type I error levels are not guaranteed.

Fig. S6. Cytoprotective properties of compounds 1-4 used at the concentration 0.01 mg/mL 60 mM against 2,2'-azobis(2-methylpropionamidine) dihydrochloride (AAPH)-induced oxidative hemolysis (means ± SD, n=12-13). Values denoted with identical letters do not differ significantly

Table S2

Scaled cytoprotective activity under oxidative stress for the higher concentration (0.01 mg/mL).

Compound	Measured cytprotective activity [%]	Scaled cytoprotective activity [%]
Trolox	39.42	86.00
1	17.81	38.85
2	28.20	61.52
3	37.51	81.83
4	32.16	70.16

Materials and methods

Human erythrocyte preparation

All methods were carried out following relevant guidelines and regulations, and the Bioethics Committee approved all experimental protocols for Scientific Research at the Medical University of Poznań (agreement no. ZP/2867/D/21). Human RBC concentrates were purchased from Blood Bank in Poznań without any contact with blood donors.

The erythrocytes were washed three times (3000 rpm, 10 min, +4°C) in 7.4 pH phosphate buffered saline (PBS: 137 mM NaCl, 2.7 mM KCl, 10 mM Na_2HPO_4, 1.76 mM KH_2PO_4) supplemented with 10 mM glucose. After washing, RBC were suspended in the PBS buffer at $1.65x10^9$ cells/mL, stored at +4°C, and used within 5 h.

Hemolytic assay

The cytotoxic activity of the compounds tested was determined by a standard hemolytic assay according to Mrówczyńska and Hägerstrand [1]. Briefly, RBC ($1.65x10^8$ cells/mL, ~1.5% hematocrit) were incubated in PBS buffer (7.4 pH) supplemented with 10 mM glucose and containing compounds tested in different concentrations (0.1 mg/mL) for 60 min at 37°C under shaking. Samples with RBC incubated in PBS without compounds tested were taken as the negative control. Each sample was repeated three times, and the experiments were repeated 3 times (n=9) with RBC from different donors. After incubation, the RBC suspensions were centrifuged (3000 rpm, 10 min), and the degree of hemolysis was estimated by measuring the absorbance (Ab) of the supernatant at 540 nm. The results were expressed as a percentage (%) of hemolysis which was determined using the following equation:

$$\text{hemolysis } \% = (\text{sample Ab}/\text{positive control Ab}) \times 100$$

were positive control is Ab value of supernatant obtained from samples with RBC incubated in ice-cold H_2O.

Inhibition of free radical-induced hemolysis

RBC ($1.65x10^8$ cells/mL, ~1.5% hematocrit) were incubated in PBS buffer (pH 7.4) supplemented with 10 mM glucose and containing compounds tested (0.1 mg/mL) for 20 min at 37°C under shaking. After pre-incubation, 2,2'-azobis(2-methylpropionamidine) dihydrochloride (AAPH) was added at the final concentration of 60 mM. Samples were incubated for the next 4 h at 37°C under shaking. Erythrocytes incubated in PBS only and in the presence of AAPH, were taken as the negative and positive controls, respectively. After incubation, the erythrocyte suspensions were centrifuged (4000 rpm, 5 min, +4° C), and the degree of hemolysis was determined by measuring the absorbance (Ab) of the supernatant at 540 nm in a spectrophotometer. The percentage of inhibition was calculated using the following equation:

$$\text{Inhibition of hemolysis } (\%) = 100 - [(Ab_{sample}/Ab_{AAPH}) \times 100\%]$$

where Ab_{sample} is the absorbance value of supernatant obtained from samples incubated with compounds tested in the presence of AAPH, and Ab_{AAPH} is the absorbance of supernatant obtained from samples with AAPH without compounds tested. Each sample was made at least in triplicate, and the results are presented as a mean value ± SD (n=12-13) value of three independent experiments with RBC from different donors.

[1] Lucyna Mrówczyńska and Henry Hägerstrand, “Platelet-Activating Factor Interaction with the Human Erythrocyte Membrane,” Journal of Biochemical and Molecular Toxicology 23, no. 5 (September 2009): 345–48, https://doi.org/10.1002/jbt.20297.
