# Supplementary material for: Artificial Intelligence in Decrypting Cytoprotective Activity under Oxidative Stress from Molecular Structure
Source: Int J Mol Sci. 2023 Jul 12;24(14):11349. doi: 10.3390/ijms241411349 (PMC10379162; doi:10.3390/ijms241411349)
Supplement: Supplementary file 1 [file ijms-24-11349-s001.zip › Synthesis and spectroscopy.pdf]

Materials and Methods

Instrumentation and Chemicals

The melting points (mp) were obtained with a Büchi SMP-20 apparatus. ^{1}H NMR and ^{13}C NMR spectra were recorded on a Varian 300/400 spectrometer with DMSO-d_6 as the solvent and TMS as the internal standard. Chemical shifts are reported in ppm (parts per million) values. EI mass spectra were measured on Bruker 320MS/450GC mass spectrometer. FT-IR spectra were recorded on Nicolet iS 5 (KBr pellets). TLC analysis was used using Sigma-Aldrich silica gel 60 plates with a fluorescent indicator (254 nm) and visualized with UV. All chemicals or reagents used for syntheses were commercially available.

Synthesis of Indole Derivatives

A typical procedure for the synthesis of compounds 1-4.

A solution of gramine (1 mmol) and the appropriate uracil derivative (1 mmol) in 5 mL of DMF was heated under reflux for 2–4 h. After completion of the reaction, as indicated by TLC (chloroform: methanol 5:1), a solution was moved to a separatory funnel and 5 mL of water was added. The mixture was extracted with diethyl ether (3 x 15 mL), then the combined organic layer was washed with water (100 mL) and brine (50 mL), dired (Na_2SO_4) and concentrated under reduced pressure. New compounds were recrystallized from toluene.

*1-((1H-indol-3-yl)methyl)-5-fluoropyrimidine-2,4(1H,3H)-dione (**1**)*

Light yellow powder (43 mg, 17%); m.p. 210-213°C; ^{1}H NMR (400 MHz, DMSO-d_6): δ 11.75 (s, 1H), 11.17 (s, 1H), 8.09 (d, *J* = 6.7 Hz, 1H), 7.66 (ddt, *J* = 7.9, 1.3, 0.7 Hz, 1H), 7.50 (d, *J* = 2.5 Hz, 1H), 7.39 (dt, *J* = 8.2, 0.9 Hz, 1H), 7.12 (ddd, *J* = 8.2, 7.0, 1.2 Hz, 1H), 7.02 (ddd, *J* = 8.0, 7.0, 1.1 Hz, 1H), 4.97 (s, 2H); ^{13}C NMR (101 MHz, DMSO-d_6): δ 157.30, 149.60, 139.64, 136.29, 129.23, 126.01, 121.56, 121.07, 119.13, 118.53, 111.70, 109.32, 42.28; IR (KBr): 3346, 3025, 1700, 1678, 1388, 1234, 1099, 751 cm^{-1}; EI-MS (m/z, % int.): 259 (M^{+}, 6), 130 (100).

*1-((1H-indol-3-yl)methyl)-6-phenyl-2-thioxo-2,3-dihydropyrimidin-4(1H)-one (**2**)*

Beige solid (32 mg, 10%); m.p. 190-192°C; ^{1}H NMR (400 MHz, DMSO-d_6) δ 12.54 (s, 1H), 10.72 (dd, *J* = 8.2, 2.4 Hz, 1H), 7.49–7.42 (m, 5H), 7.29–7.24 (m, 1H), 7.18 (dd, *J* = 8.0, 5.2 Hz, 1H), 7.05–6.98 (m, 1H), 6.87 (ddd, *J* = 7.9, 6.9, 1.0 Hz, 1H), 5.76 (s, 1H), 3.54 (s, 2H);

^{13}C NMR (101 MHz, DMSO-d_6) 174.37, 161.67, 150.16, 136.03, 131.64, 129.91, 128.52, 128.26, 128.21, 126.60, 122.58, 120.83, 118.18, 118.08, 114.54, 112.32, 112.25, 111.22, 20.70; IR (KBr): 3410, 2129, 2887, 1649, 1554, 1455, 1217, 743, 700 cm^{-1}; EI-MS (m/z, % int.): 333 (M$^+$, 100), 130 (8).

*1-((1H-indol-3-yl)methyl)-6-propyl-2-thioxo-2,3-dihydropyrimidin-4(1H)-one (**3**)*

Orange solid (70 mg, 23%); m.p.177-180°C; ^1H NMR (400 MHz, DMSO-d_6) δ 12.40 (s, 1H), 11.01 (t, *J* = 4.7 Hz, 1H), 7.86 (ddt, J = 7.9, 1.4, 0.7 Hz, 1H), 7.44 (d, *J* = 2.5 Hz, 1H), 7.33 (dt, *J* = 8.0, 1.0 Hz, 1H), 7.17 (dtd, *J* = 8.5, 1.3, 0.7 Hz, 1H), 7.08 – 7.02 (m, 1H), 6.98 (ddd, *J* = 8.0, 7.0, 1.2 Hz, 1H), 5.64 (s, 2H), 2.36–2.30 (m, 2H), 1.60–1.48 (m, 1H), 0.86 (t, *J* = 7.3 Hz, 2H); ^{13}C NMR (101 MHz, DMSO-d_6) δ 176.47, 160.35, 155.18, 135.61, 128.90, 128.21, 126.30, 120.91, 119.72, 118.56, 111.32, 102.24, 40.37, 32.93, 20.52, 13.28; IR (KBr): 3399, 3134, 2963, 1680, 1537, 1457, 1319, 1218, 743 cm^{-1}; EI-MS (m/z, % int.): 299 (M$^+$, 22), 130 (100).

*1-((1H-indol-3-yl)methyl)-5-propyl-2-thioxo-2,3-dihydropyrimidin-4(1H)-one (**4**)*

White solid (30 mg, 20%); m.p. 187-190°C; ^1H NMR (400 MHz, DMSO-d_6) δ 12.54 (s, 1H), 11.19 (s, 1H), 7.78 (dq, *J* = 8.0, 0.7 Hz, 1H), 7.70 (d, *J* = 1.0 Hz, 1H), 7.56 (d, *J* = 2.6 Hz, 1H), 7.39 (dt, *J* = 8.1, 0.9 Hz, 1H), 7.11 (ddd, *J* = 8.2, 7.0, 1.2 Hz, 1H), 7.01 (ddd, *J* = 8.0, 7.0, 1.0 Hz, 1H), 5.58 (s, 2H), 2.17–2.09 (m, 2H), 1.42–1.30 (m, 2H), 0.77 (t, *J* = 7.3 Hz, 3H); ^{13}C NMR (101 MHz, DMSO-d_6) δ 174.73, 160.53, 140.97, 136.22, 126.31, 126.02, 121.54, 119.08, 119.02, 118.96, 111.72, 109.01, 48.00, 28.14, 20.80, 13.34.; IR (KBr): 3269, 3079, 2955, 1681, 1497, 1323, 1255, 1167, 738 cm^{-1}; EI-MS (m/z, % int.): 299 (M$^+$, 11), 130 (100).

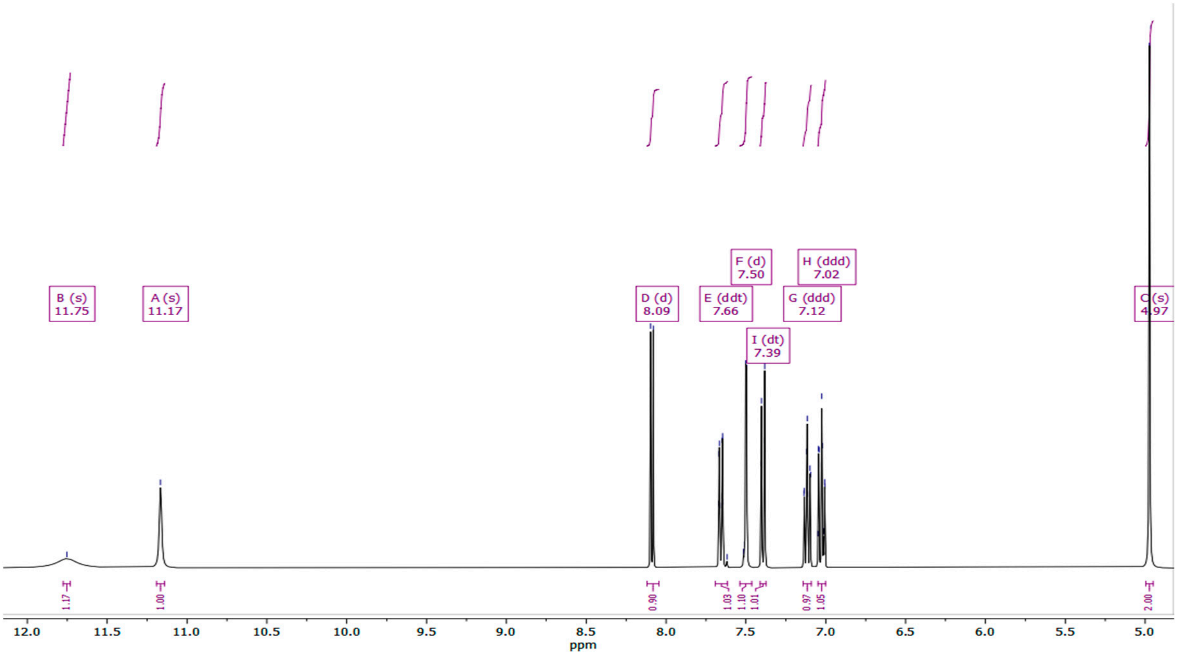

Figure S1a. ^{1}H NMR spectrum of compound **1**

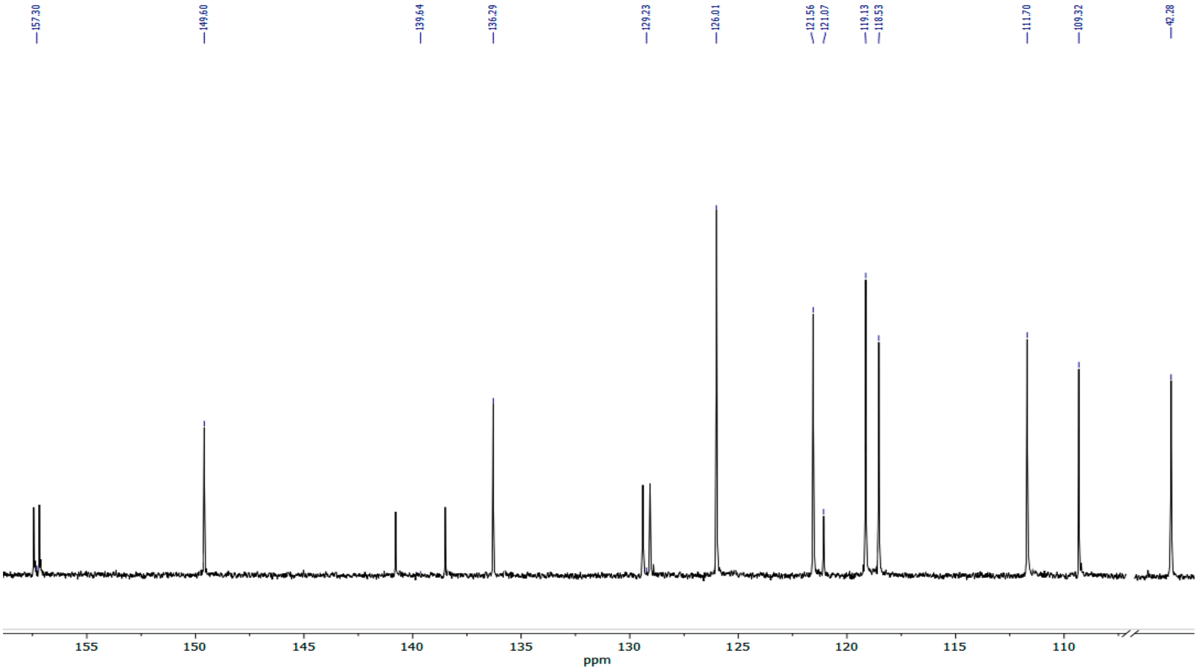

Figure S1b. ^{13}C NMR spectrum of compound **1**

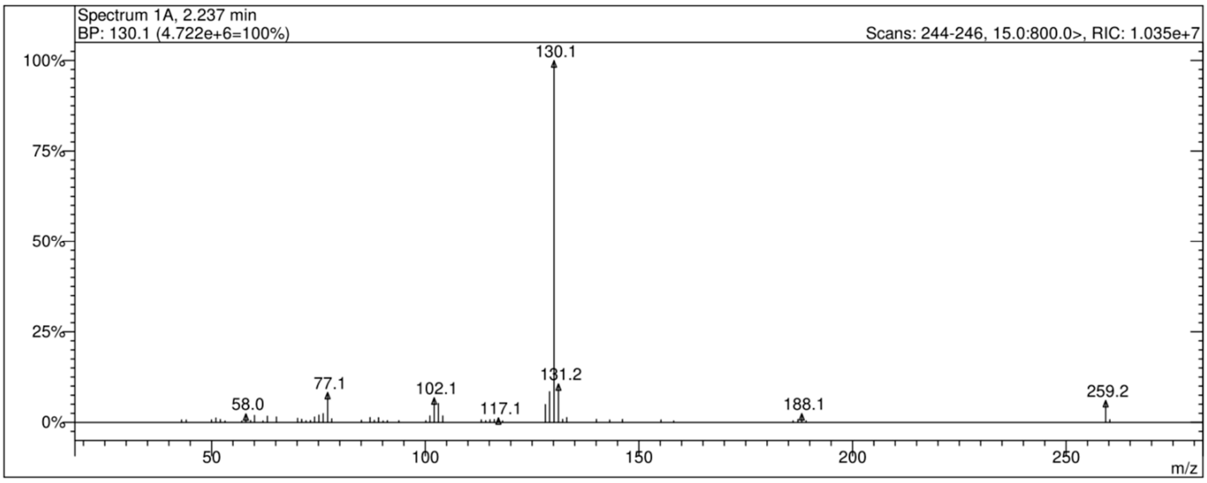

Figure S1c. EI-MS spectrum of compound **1**

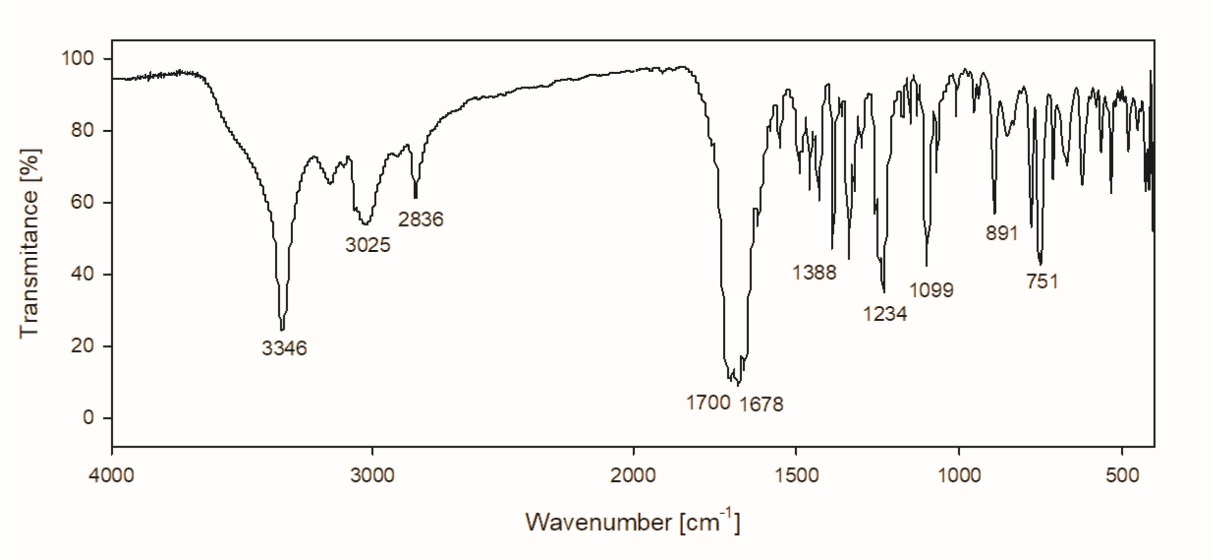

Figure S1d. IR spectrum of compound **1**

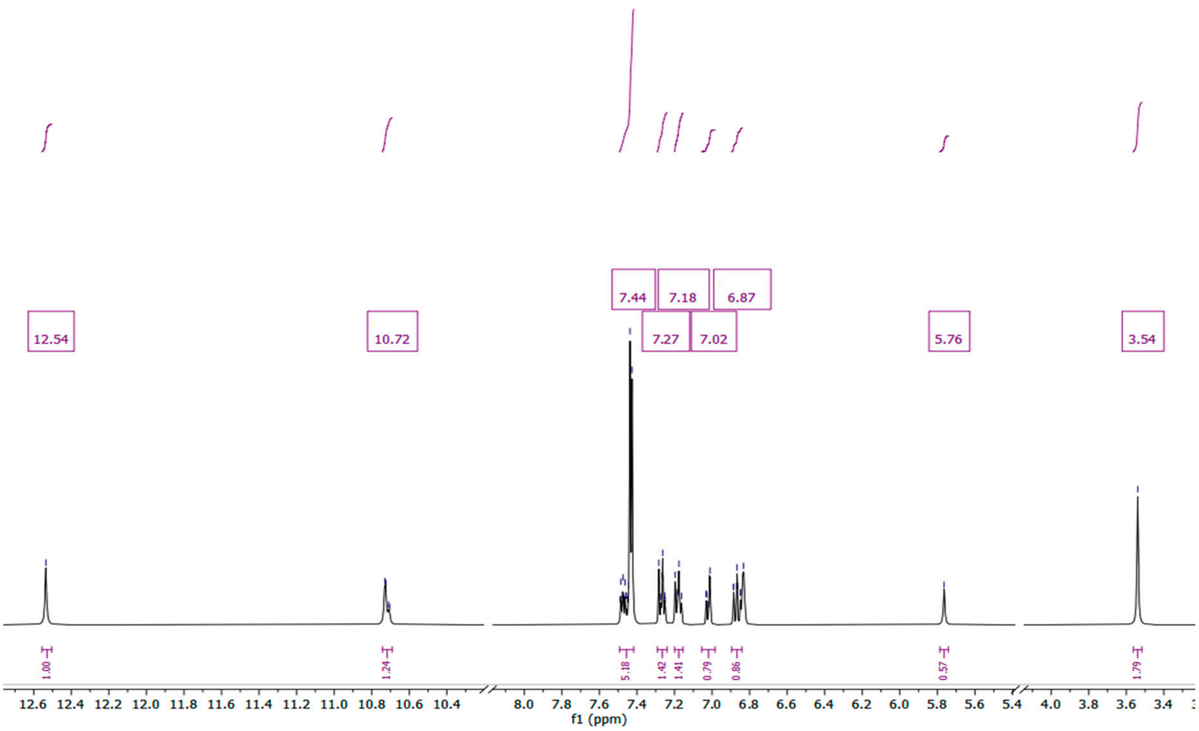

Figure S2a. ^{1}H NMR spectrum of compound **2**

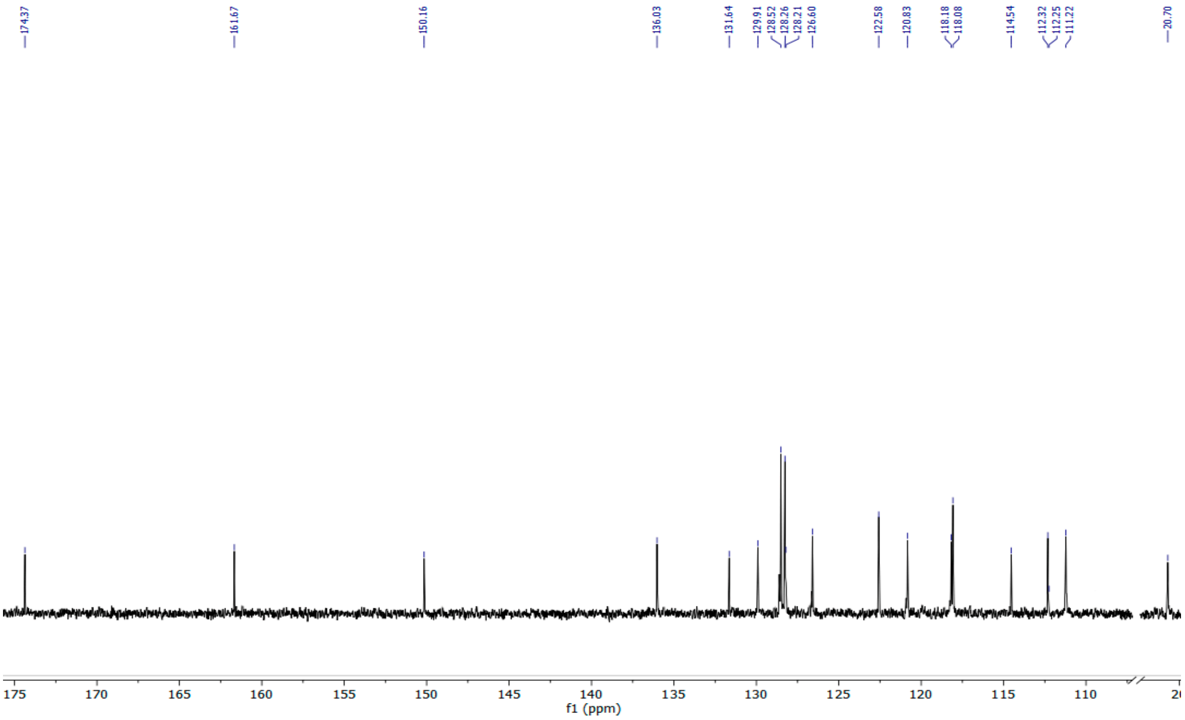

Figure S2b. ^{13}C NMR spectrum of compound **2**

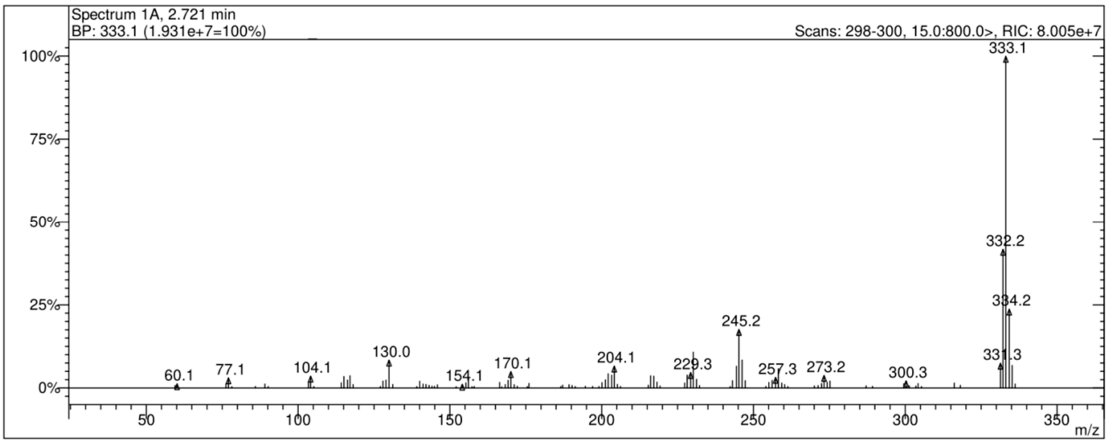

Figure S2c. EI-MS spectrum of compound **2**

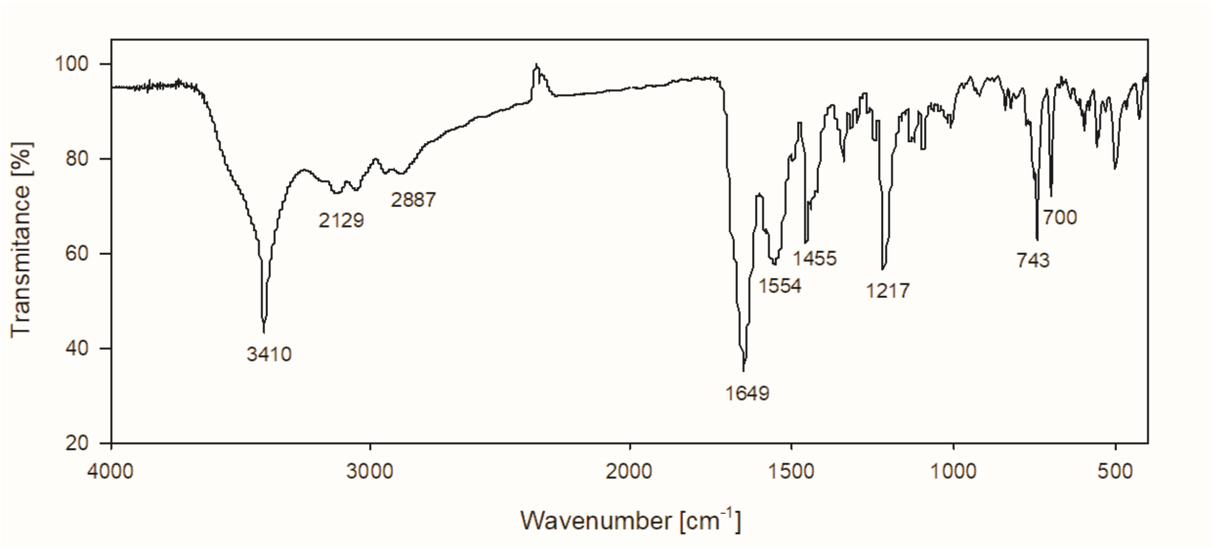

Figure S2d. IR spectrum of compound **2**

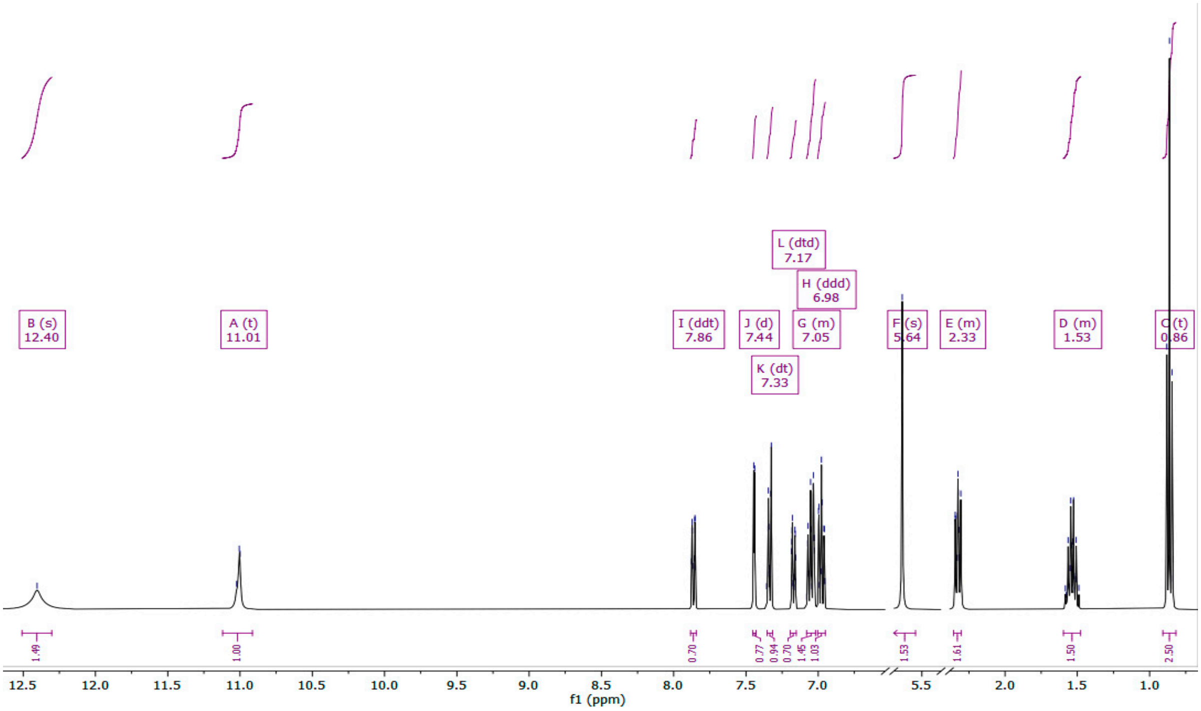

Figure S3a. ^{1}H NMR spectrum of compound **3**

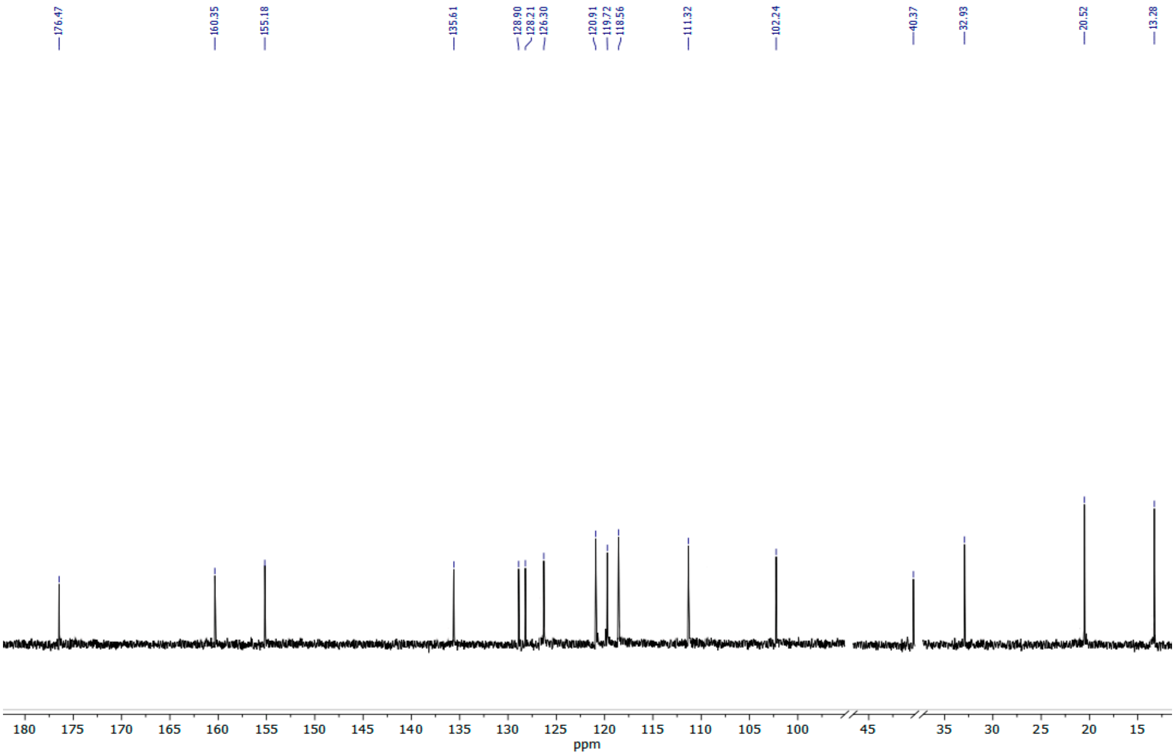

Figure S3b. ^{13}C NMR spectrum of compound **3**

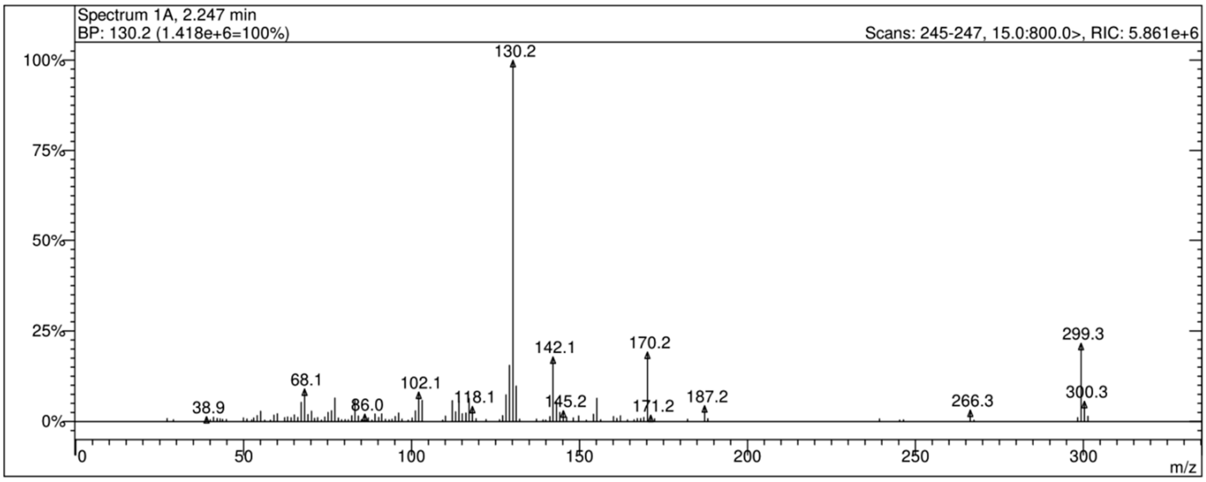

Figure S3c. EI-MS spectrum of compound **3**

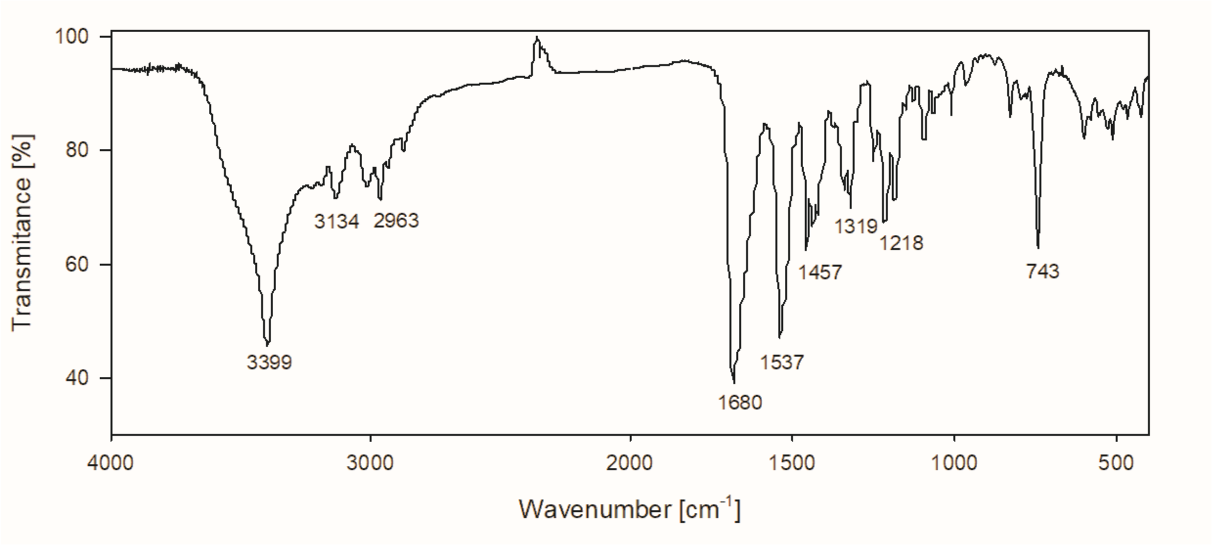

Figure S3d. IR spectrum of compound **3**

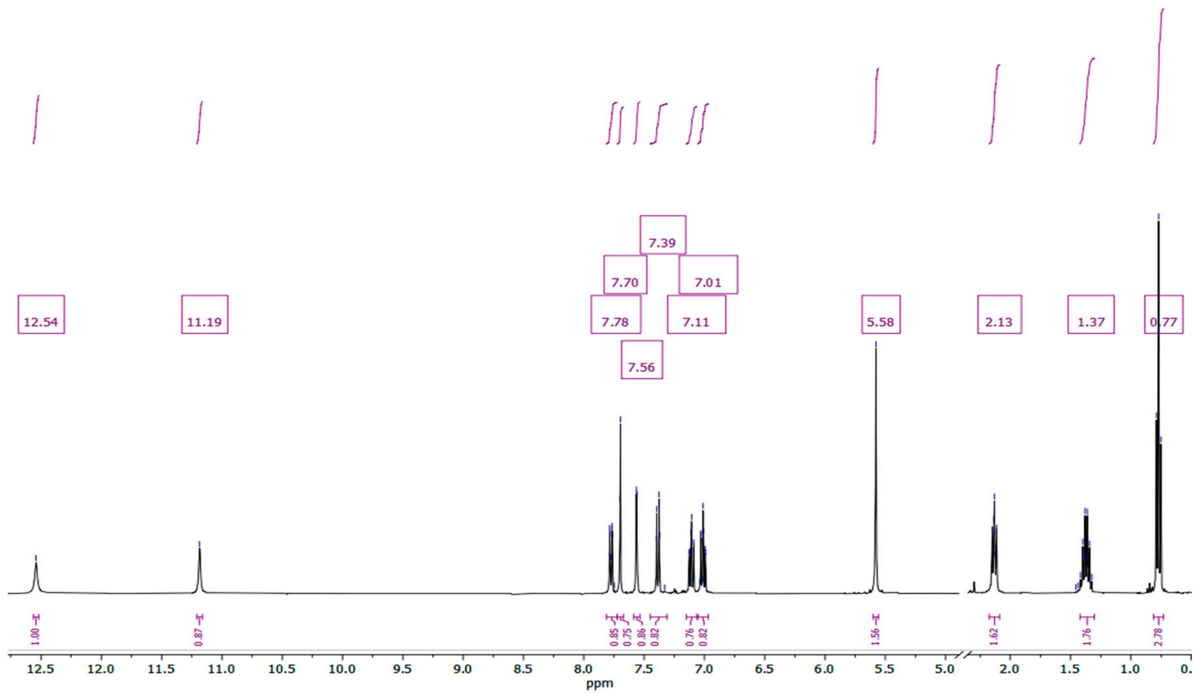

Figure S4a. ^{1}H NMR spectrum of compound **4**

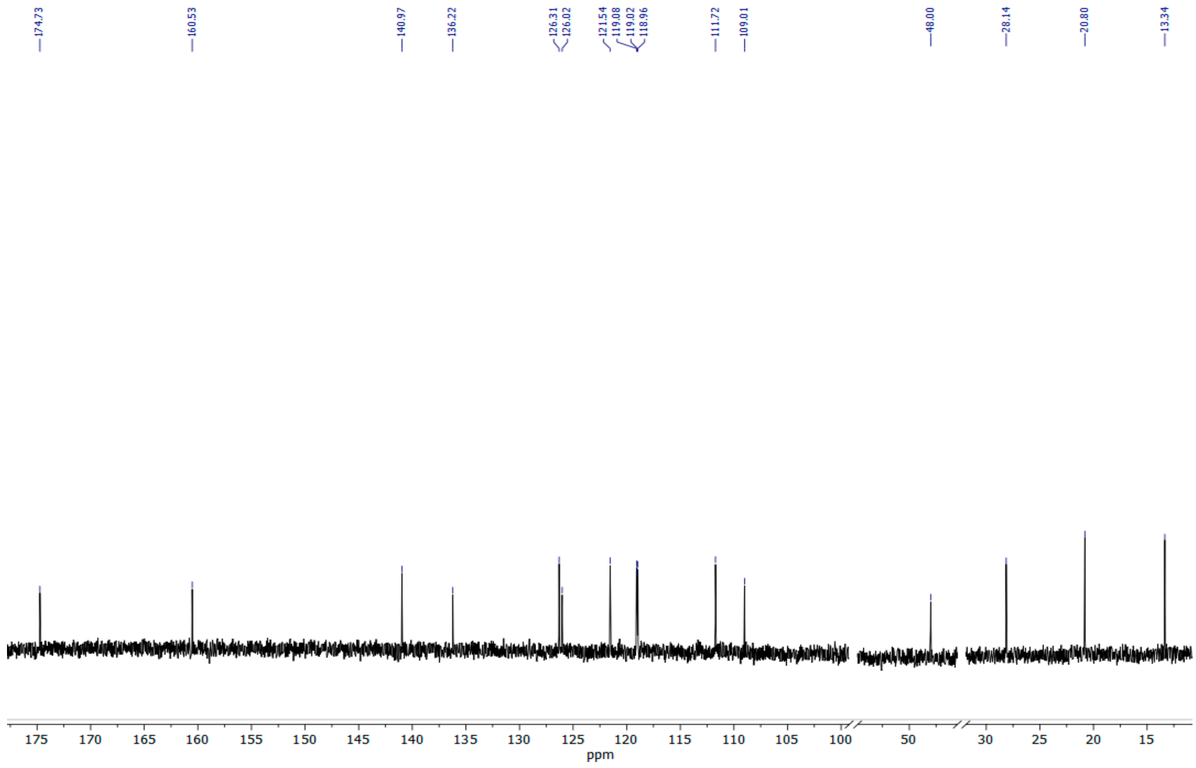

Figure S4b. ^{13}C NMR spectrum of compound **4**

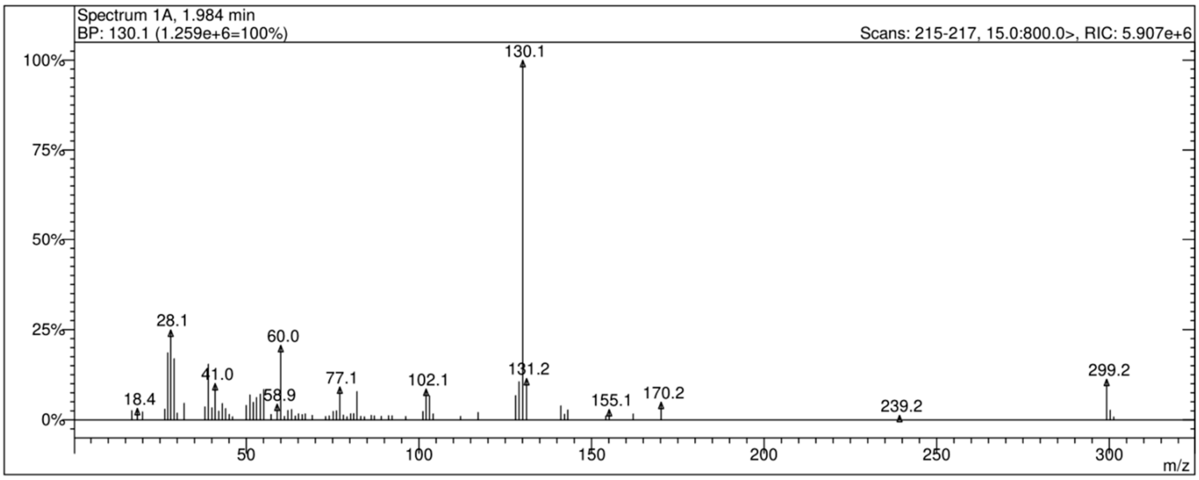

Figure S4c. EI-MS spectrum of compound **4**

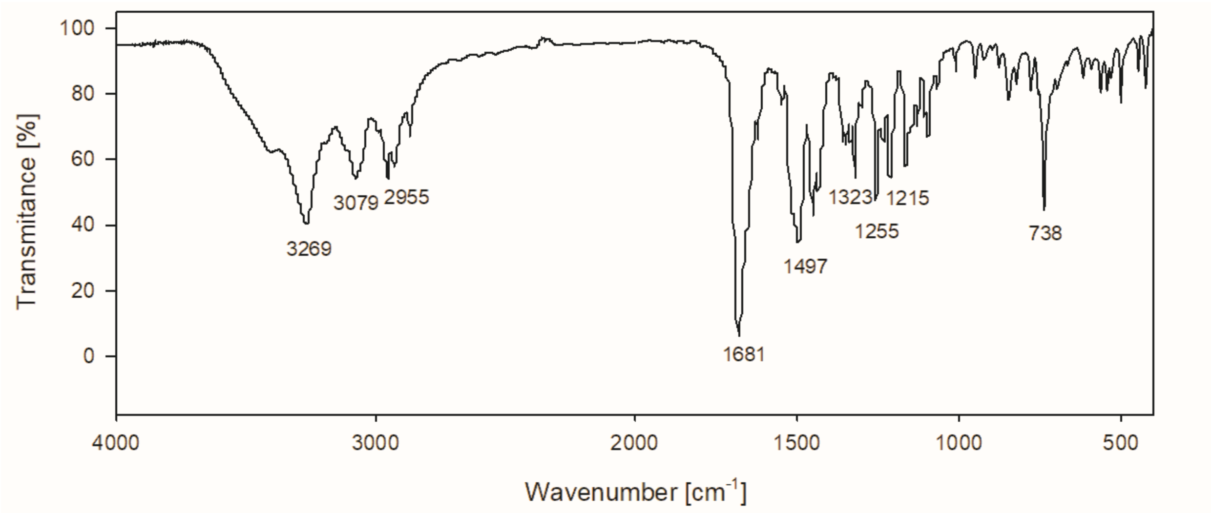

Figure S4d. IR spectrum of compound **4**
