# Supplementary figures and images for: Artificial Intelligence in Decrypting Cytoprotective Activity under Oxidative Stress from Molecular Structure
Source: Int J Mol Sci. 2023 Jul 12;24(14):11349. doi: 10.3390/ijms241411349 (PMC10379162; doi:10.3390/ijms241411349)

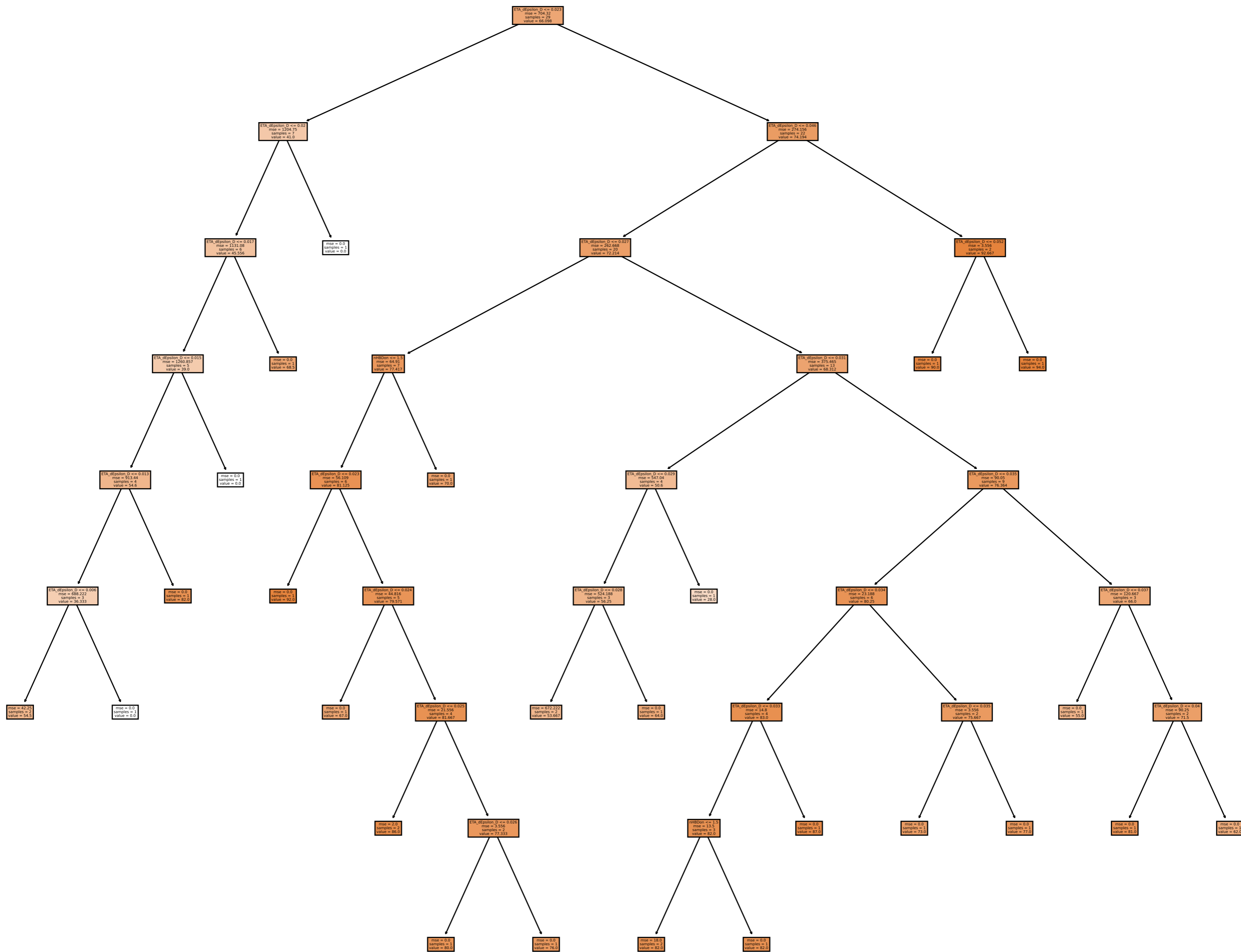

Supplement: Supplementary file 1 [file ijms-24-11349-s001.zip › The_final_regression_predictive_model_decision_tree_scheme.pdf]
